# Integral equation models for solvent in macromolecular crystals

**DOI:** 10.1063/5.0070869

**Published:** 2022-01-04

**Authors:** Jonathon G. Gray, George M. Giambaşu, David A. Case, Tyler Luchko

**Affiliations:** 1 Department of Chemistry and Chemical Biology, Rutgers University, Piscataway, New Jersey 08854, USA; 2 Institute for Quantitative Biomedicine, Rutgers University, Piscataway, New Jersey 08854, USA; 3 Department of Physics and Astronomy, California State University, Northridge, California 91330, USA

## Abstract

The solvent can occupy up to ∼70% of macromolecular crystals, and hence, having models that predict solvent distributions in periodic systems could improve the interpretation of crystallographic data. Yet, there are few implicit solvent models applicable to periodic solutes, and crystallographic structures are commonly solved assuming a flat solvent model. Here, we present a newly developed periodic version of the 3D-reference interaction site model (RISM) integral equation method that is able to solve efficiently and describe accurately water and ion distributions in periodic systems; the code can compute accurate gradients that can be used in minimizations or molecular dynamics simulations. The new method includes an extension of the Ornstein–Zernike equation needed to yield charge neutrality for charged solutes, which requires an additional contribution to the excess chemical potential that has not been previously identified; this is an important consideration for nucleic acids or any other charged system where most or all the counter- and co-ions are part of the “disordered” solvent. We present several calculations of proteins, RNAs, and small molecule crystals to show that x-ray scattering intensities and the solvent structure predicted by the periodic 3D-RISM solvent model are in closer agreement with the experiment than are intensities computed using the default flat solvent model in the refmac5 or phenix refinement programs, with the greatest improvement in the 2 to 4 Å range. Prospects for incorporating integral equation models into crystallographic refinement are discussed.

## I. INTRODUCTION

Ions and water molecules have been long known to play crucial roles in governing biomolecular stability and function. Elucidating how ions and water molecules distribute themselves around the solutes should provide valuable insights into how these molecules function and also provide experimental tests for theoretical predictions. However, there are a few methods that directly probe the distributions of ions and water molecules around macromolecules. In solution, excess numbers of water molecules and ions around a macromolecule can be obtained using atomic emission spectroscopy,^1,2^ small-angle x-ray scattering,^3–6^ or measurements of partial molar volumes.^7–10^ These techniques, however, give relatively little information about the distribution of water molecules and ions in the vicinity of a biomolecule.

In principle, much more detailed information is available from x-ray diffraction studies on biomolecular crystals, and it is common to include some number of “bound” (or localized) water molecules and ions in a refined atomic model that has been optimized to fit observed scattering intensities. These locations are typically identified as features in a difference electron density map that satisfy criteria for both intensity (percent occupation) and geometry. However, the “bound” solvent molecules generally make up only a small fraction of the total solvent; the remainder is typically modeled as a flat distribution, usually with density and B-factor components that are adjusted to optimize the fit of the total model to observed intensities. The limitations of such a flat-density model are thought to contribute to the “R-factor gap,” which reflects the nearly universal observation that differences between computed and observed intensities in macromolecular crystallography are much greater than the experimental uncertainties, prompting searches for better models.^11^

In this paper, we develop and apply a novel integral equation model, the 3D-reference interaction site model (3D-RISM), to predict the solvent distribution in both small molecule and macro-molecular crystals of proteins and nucleic acids. We present results from a newly developed periodic version of the existing non-periodic 3D-RISM in Amber.^12–14^ Particular attention is paid to the way in which charged solutes are handled to ensure the electroneutrality of the entire unit cell, that is, to ensure that the distribution of ions in the solvent counterbalances the net charge of the solute. The 3D-RISM has been used in non-periodic systems to predict the location of site bound water molecules and ions and quantities reporting on the diffuse and territorial binding modes of solvent particles (ion counting and scattering profiles) and to give quantitative energetics of solvation or small molecule binding to biomolecules.^15–20^ Here, we explore the application of similar ideas to crystalline systems.

## II. REFERENCE INTERACTION SITE MODEL FOR PERIODIC SYSTEMS

The core principle of the RISM is to find the single particle density distributions that minimize the excess chemical potential in response to an external potential arising from a molecular solute. The basic idea and the approximations involved have been discussed many times,^12,17^ and we only give a brief summary here. In principle, the distribution of solvent molecules around a (fixed) solute is a six-dimensional quantity, describing the translation and orientations of the solvent molecules. Such an approach is used in molecular Ornstein–Zernike^21^ and molecular density functional theories.^22^ In contrast, the 3D-RISM formalism reduces these to three-dimensions by decomposing polyatomic solvents (such as water molecules) into atomic contributions such that the resulting solvent density distributions contain only a spatial dependence, $\rho_{\gamma }\left(r\right)$, and can be represented by scalar densities on 3D grids. Here, the solvent index *γ* would range over H and O sites in water and over mobile atomic cations, such as Na^+^ and Cl^−^.

An Ornstein–Zernike-like equation relates the total correlation function, $h_{\gamma }\left(r\right)=g_{\gamma }\left(r\right)-1$, and direct correlation function, $c_{\gamma }\left(r\right)$, through a convolution (denoted by *) as follows:

$$h_{\gamma }^{\text{OZ}}\left(r\right)=\underset{\alpha }{\sum }c_{\alpha }\left(r\right)*\chi_{\alpha \gamma }\left(r\right).$$ (1)

Here, $\chi_{\alpha \gamma }\left(r\right)=\omega_{\alpha \gamma }\left(r\right)+\rho_{\alpha }h_{\alpha \gamma }\left(r\right)$ is the site–site solvent susceptibility of solvent sites *α* and *γ* and describes the orientationally averaged bulk properties of the solvent, where $\omega_{\alpha \gamma }\left(r\right)$ is an intramolecular correlation matrix, *ρ*_*α*_ is the bulk number density, and $h_{\alpha \gamma }\left(r\right)$ is the total correlation function. These values are pre-computed (generally by a “1D-RISM” approach) for the reference solvent using the dielectrically consistent RISM (DRISM) integral equation.^23,24^ As in earlier work,^12,13^ entities with two subscripts, such as $h_{\alpha \gamma }\left(r\right)$, refer to solvent–solvent interactions, whereas entities with a single subscript, such as $h_{\gamma }^{OZ}\left(r\right)$, refer to solvent site *γ* at point **r** on the three-dimensional grid surrounding the solute.

Equation (1) is augmented by a 3D closure relation as follows:

$$h_{\gamma }^{\text{closure}}\left(r\right)=\exp\left\{-\beta u_{\gamma }\left(r\right)+h_{\gamma }^{\text{OZ}}\left(r\right)-c_{\gamma }\left(r\right)+b_{\gamma }\left(r\right)\right\}-1,$$ (2)

where $b_{\gamma }\left(r\right)$ is the bridge function, which is only known as an infinite series of functionals and is always subject to some approximation.^25^ Among the many closure relations that have been developed, in this work, we use a family of closures related to the hypernetted chain (HNC) closure,^26^ where the bridge function is simply set to zero. The HNC produces good results for ionic^27–29^ and polar systems^30,31^ and has an exact closed form expression for the excess chemical potential.^32^ Since HNC solutions are often difficult to converge, one can use intermediaries such as the so-called partial series expansion of order-*n* (PSE-*n*)^33^ of the HNC as a Taylor series expansion when the exponent in Eq. (2) is positive,

$$h_{\gamma }^{\text{PSE}-n}\left(r\right)=\left\{\begin{array}{cc} \exp\left\{t_{\gamma }\left(r\right)\right\}-1, & \;t_{\gamma }\left(r\right)<0,\\ \overset{n}{\underset{i=1}{\sum }}\frac{{t_{\gamma }\left(r\right)}^{i}}{i!}, & \;t_{\gamma }\left(r\right)\geq 0, \end{array}\right.$$ (3)

$$t_{\gamma }\left(r\right)=-\beta u_{\gamma }\left(r\right)+h_{\gamma }^{\text{OZ}}\left(r\right)-c_{\gamma }\left(r\right),$$

where the HNC is the limiting case as *n* → *∞*. As for the HNC, the PSE-*n* family of closures have an exact closed form expression for the chemical potential. The form of this approximation has a major impact on the convergence of calculations and on resulting thermodynamic quantities and correlation functions.

The goal of the self-consistent 3D-RISM procedure can be viewed as finding a direct correlation function $c_{\gamma }\left(r\right)$ such that $h_{\gamma }^{\text{OZ}}$ and $h_{\gamma }^{\text{closure}}$ become identical at all grid points to within some (fairly tight) tolerance. In existing non-periodic implementations, the convolution required in Eq. (1) is carried out via fast Fourier transform (FFT) in a rectangular box surrounding the solute, and additional terms that account for the solvent outside of the artificial box are added to this. Key changes for crystals are that the electrostatic and Lennard-Jones potentials that appear in Eq. (3) need to take periodic boundary conditions into account and that some special considerations are needed, when the solute has a net charge, to ensure charge neutrality for each unit cell. While our implementation for non-periodic boundaries uses direct and treecode summation,^12,14^ periodic methods [e.g., particle mesh Ewald (PME) and Ewald summation] have been used before to synthesize the long-range electrostatic potential on a 3D grid.^34,35^ These approaches generally assume infinite dilution of the solute and employ corrections to capture the long-range, open-boundary behavior of the solvent and net charge of the solute when calculating the excess chemical potential. Because of these long-range corrections, previous methods employing PME or Ewald summation are not suitable for crowded periodic systems, such as crystals. In contrast, we use periodic boundary conditions throughout the method described in Secs. II A and II B.

### A. Constructing the periodic solute potential

The closure functional equation requires the mapping of the solute potential onto regular grids that covers the entire unit cell with one potential grid for each type of solvent site encompassing both Lennard-Jones and electrostatic components. Mapping the electrostatic potential follows the smooth PME (SPME) procedure used in molecular dynamics simulations^36,37^ although the grid spacing is smaller, typically 0.5 Å. Lennard-Jones interactions between solute atoms and all solvent types are calculated at each grid point using a distance cutoff (the default is 9 Å) and the minimum-image convention. The same convention is used for the short-range part of the electrostatic potential where the bare Coulomb interaction is replaced by $erfc\left(\beta \left|r-r_{i}\right|\right)/\left|r-r_{i}\right|$, where **r** is the position of a solute atom and **r**_*i*_ is a point on the grid. The remaining, long-range part of the periodic Coulomb potential is solved for in the reciprocal space via fast Fourier transform (FFT) and follows the steps given below.^36,37^

1. Interpolate the solute atomic charges to the direct space Cartesian grid. The current version of the code relies on the smooth PME (SPME) approach, which uses a cardinal b-spline of order 4 or 6 to interpolate the source charge to the grid. The b-spline interpolation has a roughly Gaussian character at high polynomial orders and has the desirable trait that integration of its weights over the region of interpolation equals unity.
2. Convert the source charge grid from real space to reciprocal space using an FFT.
3. Compute the electrostatic potential and spatial derivatives (electrostatic field) on the grid using a convolution with a reciprocal space representation of the Gaussian kernel and its derivatives; in reciprocal space, the convolution is a simple multiplication, and the electrostatic interaction potential Green’s function is *k*^−2^.
4. Obtain the real space representation of the electrostatic potential and electrostatic field using an inverse FFT.

Full details of this procedure are given elsewhere.^38^

### B. Solving the 3D-RISM equations

As noted above, solving the 3D-RISM equations amounts to finding a direct correlation functional, *c*_*γ*_, for each solute site *γ* that minimizes the residual: $\Delta c_{\gamma }\left(r\right)\equiv h_{\gamma }^{\text{closure}}\left(r\right)-h_{\gamma }^{\text{O}Z}\left(r\right)$. Calculations are initialized with a guess for each *c*_*γ*_, which are chosen to be uniformly zero, although the code allows for a user-provided starting point that can accelerate convergence for systems difficult to solve. Each self-consistent cycle begins with computing $h_{\gamma }^{\text{OZ}}$ in the reciprocal space using Eq. (1), followed by a switch to the real space, where $h_{\gamma }^{\text{closure}}$ is computed using Eq. (2), and ends by modifying the current guess for *c*_*γ*_ using the modified direct inversion of the iterative subspace (MDIIS) procedure^12,39^ based upon Δ*c*_*γ*_ and a specified number of past *c*_*γ*_ solutions. This cycle is repeated until the root-mean squared residual, $\text{RMS}\left(\Delta c_{\gamma }\right)$, reaches a pre-determined threshold, which is typically 10^−10^ if gradients are needed (such as in the case of minimization or dynamics) and 10^−6^ if one just needs thermodynamic parameters or solvent distribution functions. Once convergence is obtained, there is no longer any distinction between *h*^closure^ and *h*^OZ^.

This procedure is complicated when charged solutes are used: here, one wants the solute net charge to be neutralized by the converged ion distribution of the solvent. However, a consequence of using PME is that a uniform neutralizing background charge is imposed on the system, i.e., the effective net charge of the solute is always zero if only the PME component of the potential is used. As a result, the *h*^OZ^ distribution arising from Eq. (1) will also be neutral, which is a problem when the solute charge is non-zero. We describe here a procedure modeled after that used by Kovalenko and Hirata (KH) for the non-periodic 3D-RISM,^34,39^ which modifies the OZ direct correlation function to account for this implicit background charge. We first note that the potential energy due to the solvent site *γ* interacting with a non-neutral solute is

$$u_{\gamma }\left(r\right)=u_{\gamma }^{\text{PME}}\left(r\right)-u_{\gamma }^{\text{bk}}\left(r\right)=u_{\gamma }^{\text{PME}}\left(r\right)-q_{\gamma }\phi^{\text{bk}}\left(r\right),$$ (4)

where $u_{\gamma }^{\text{PME}}\left(r\right)$ is the potential energy calculated by PME, $u_{\gamma }^{\text{bk}}\left(r\right)$ is the potential energy due to the neutralizing background charge, and $\phi^{\text{bk}}\left(r\right)$ is the background potential imposed by PME. Since $u_{\gamma }^{\text{bk}}$ represents the interaction of the solvent charge on site γ with a background charge density of infinite extent, it diverges, and we cannot directly use Eq. (4) in Eq. (2) as it stands. However, an analytic expression for $\phi^{\text{bk}}\left(r\right)$ can be found in reciprocal space: using the fact that the background charge distribution is $q^{\text{bk}}\left(r\right)=-Q_{\text{solute}}/V_{\text{cell}}$, we can write Poisson’s equation as

$${\hat{\phi }}^{\text{bk}}\left(k\right)=4\pi \frac{{\hat{q}}^{\text{bk}}\left(k\right)}{k^{2}}=-\delta \left(k\right)\frac{Q_{\text{solute}}}{V_{\text{cell}}}\frac{4\pi }{k^{2}}.$$ (5)

The restriction to *k* = 0 yields a uniformly distributed quantity in real-space but has the expected singularity at *k* = 0. Using the HNC for simplicity, Eq. (2) can then be written as

$$\begin{array}{ll} h_{\gamma }^{\text{HNC}}\left(r\right)+1 & =\exp\left[-\beta u_{\gamma }\left(r\right)+h_{\gamma }^{\text{OZ}}\left(r\right)-c_{\gamma }\left(r\right)\right]\\ & =\exp\left[-\beta u_{\gamma }^{\text{PME}}\left(r\right)+h_{\gamma }^{\text{OZ}}\left(r\right)-{\tilde{c}}_{\gamma }\left(r\right)\right], \end{array}$$ (6)

where we have grouped the background charge contribution with $c_{\gamma }\left(r\right)$ to define a renormalized direct correlation function as follows:

$${\tilde{c}}_{\gamma }\left(r\right)=c_{\gamma }\left(r\right)-\beta q_{\gamma }\phi^{\text{bk}}\left(r\right).$$ (7)

The Ornstein–Zernike equation, Eq. (1), is then

$$\begin{array}{ll} h_{\gamma }^{\text{OZ}}\left(r\right) & =\underset{\alpha }{\sum }c_{\alpha }\left(r\right)*\chi_{\alpha \gamma }\left(r\right)\\ & =\underset{\alpha }{\sum }\left[{\tilde{c}}_{\alpha }\left(r\right)*\chi_{\alpha \gamma }\left(r\right)+\beta u_{\alpha }^{\text{bk}}\left(r\right)*\chi_{\alpha \gamma }\left(r\right)\right]. \end{array}$$ (8)

Taking the Fourier transform, which we denote by $\hat{\cdot }$, we obtain

$$\begin{array}{ll} \underset{\alpha }{\sum }\beta {\hat{u}}_{\alpha }^{\text{bk}}\left(k\right){\hat{\chi }}_{\alpha \gamma }\left(k\right) & =-\underset{\alpha }{\sum }\beta \delta \left(k\right)q_{\alpha }\frac{Q_{\text{solute}}}{V_{\text{cell}}}\frac{4\pi }{k^{2}}{\hat{\chi }}_{\alpha \gamma }\left(k\right)\\ & =-4\pi \beta \frac{Q_{\text{solute}}}{V_{\text{cell}}}\delta \left(k\right)\underset{k\to 0}{\text{lim}}\underset{\alpha }{\sum }\frac{q_{\alpha }}{k^{2}}{\hat{\chi }}_{\alpha \gamma }\left(k\right)\\ & \equiv {\hat{h}}_{\gamma }^{\text{bk}}\delta \left(k\right). \end{array}$$ (9)

This depends only on *Q*_solute_, *V*_cell_, and the properties of the bulk solvent and evaluates to a constant when going back to real space. Even though ${\hat{\phi }}^{\text{bk}}\left(k\right)$ in Eq. (5) has a singularity at *k* = 0, ${\hat{h}}_{\gamma }^{\text{bk}}$ in Eq. (9) is finite. In practice, we use a polynomial interpolation procedure based on Neville’s algorithm to numerically extrapolate values at finite *k* in Eq. (9) to the *k* = 0 limit.

**FIG. 1.**
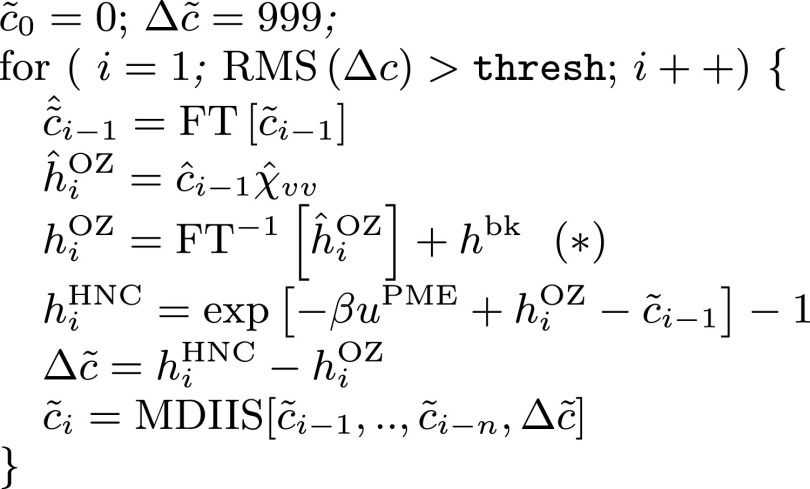
Pseudo-code for an algorithm to solve the 3D-RISM equations using $\tilde{c}$ and a “shift” in *h*^OZ^ (starred line). The code assumes HNC closure for simplicity; the other starting estimates for ${\tilde{c}}_{0}$ may be used, such as the result from a previous step of minimization or MD. FT is the Fourier transform, MDIIS is a version of the direct inversion of the iterative subspace,^12,39^ and thresh is a convergence threshold. For a neutral solute, or for pure water as a solvent, *h*^bk^ is zero.

Modifying *h*^OZ^(***r***) by a constant would seem to yield a distribution function *g*(***r***) ≡ *h*(***r***) + 1 that is not zero inside solute atoms. However, during the self-consistent cycle, this shift is immediately followed by an application of the closure relation, Eq. (6), with a contribution exp[−*βu*(***r***)] that serves to prevent solvent species from being close to solute atoms, as discussed in Refs. 34 and 39. Solving Eqs. (6) and (8), rather than the original equations (1) and (2), implies that the renormalized ${\tilde{c}}_{\gamma }\left(r\right)$ is used throughout the algorithm in Fig. 1. By doing so, the solvent distribution will exactly neutralize the solute charge even though we only use the neutralized potential energy, $u_{\gamma }^{\text{PME}}\left(r\right)$.

In the end, the procedure for charged solutes is only slightly modified from that used for neutral solutes: we use $\tilde{c}$ rather than *c* [Eq. (7)] and “shift” $h_{\gamma }^{\text{OZ}}$ by $h_{\gamma }^{\text{bk}}$ [Eq. (9)]. The pseudo-code for this process is given in Fig. 1.

### C. Computing the excess chemical potential for a shifted OZ model

When the algorithm in Fig. 1 is complete, we have the total correlation function for the charged solute, $h_{\gamma }\left(r\right)$, but the direct correlation function used in the calculation, ${\tilde{c}}_{\gamma }\left(r\right)$, contains the background potential energy. This must be accounted for when calculating the excess chemical potential. Here, we follow Kovalenko and Hirata^34^ and start by considering the Kirkwood charging formula for the excess chemical potential, but we use $u_{\gamma }^{\text{PME}}\left(r,\lambda \right)$ rather than the full potential,

$$\Delta \mu =\underset{\gamma }{\sum }\rho_{\gamma }\int_{0}^{1}\int_{V_{\text{cell}}}\frac{\partial u_{\gamma }^{\text{PME}}\left(r,\lambda \right)}{\partial \lambda }g_{\gamma }\left(r,\lambda \right)\;drd\lambda ,$$ (10)

where *λ* is a coupling parameter between the solute and solvent. To find a closed form expression for Δ*μ*, we will recast the integrand in the form of an exact differential in *λ*. To begin, we consider the functional variation of the HNC closure, Eq. (6),

$$\delta h_{\gamma }\left(r\right)=-g_{\gamma }\left(r\right)\beta \delta u_{\gamma }^{\text{PME}}\left(r\right)+g_{\gamma }\left(r\right)\delta h_{\gamma }\left(r\right)-g_{\gamma }\left(r\right)\delta {\tilde{c}}_{\gamma }\left(r\right),$$ (11)

which is valid for any variation, including the variation with respect to *λ*. We can solve for the integrand in Eq. (10) using *g*_*γ*_ = *h*_*γ*_ + 1 and $\delta \left[h_{\gamma }^{2}\left(r,\lambda \right)\right]=2h_{\gamma }\left(r,\lambda \right)\delta h_{\gamma }\left(r,\lambda \right)$ to arrive at

$$\beta g_{\gamma }\left(r\right)\delta u_{\gamma }^{\text{PME}}\left(r\right)=\delta \left[\frac{{\left(h_{\gamma }\left(r\right)\right)}^{2}}{2}-{\tilde{c}}_{\gamma }\left(r\right)\right]-h_{\gamma }\left(r\right)\delta {\tilde{c}}_{\gamma }\left(r\right).$$ (12)

The first term is already in the form of an exact differential, and we use the following variation to cast the second term to a useful form:

$$\begin{array}{ll} & \delta \underset{\gamma }{\sum }\rho_{\gamma }\int_{V_{\text{cell}}}h_{\gamma }\left(r\right){\tilde{c}}_{\gamma }\left(r\right)\;dr=\int_{V_{\text{cell}}}\underset{\gamma }{\sum }\rho_{\gamma }\underset{\alpha }{\sum }\left[{\tilde{c}}_{\alpha }\left(r\right)*\chi_{\alpha \gamma }\left(r-r^{\prime }\right)\delta {\tilde{c}}_{\gamma }\left(r\right)\right.\\ & \;\;\left.+{\tilde{c}}_{\gamma }\left(r\right)\delta {\tilde{c}}_{\alpha }\left(r\right)*\chi_{\alpha \gamma }\left(r-r^{\prime }\right)\right]\\ & \;\;-\underset{\gamma }{\sum }\rho_{\gamma }h_{\gamma }^{\text{bk}}\delta {\tilde{c}}_{\gamma }\left(r\right)\;dr, \end{array}$$

where we have substituted *h*_*γ*_ using Eq. (1). As $\chi_{\alpha \gamma }\left(r-r^{\prime }\right)$ is a property of the bulk solvent and, consequently, invariant, we have

$$\begin{array}{ll} \delta \underset{\gamma }{\sum }\rho_{\gamma }\int_{V_{\text{cell}}}h_{\gamma }\left(r\right){\tilde{c}}_{\gamma }\left(r\right)\;dr & =\int_{V_{\text{cell}}}\underset{\gamma }{\sum }\rho_{\gamma }2h_{\gamma }\left(r\right)\delta {\tilde{c}}_{\gamma }\left(r\right)\\ & \;+\underset{\gamma }{\sum }\rho_{\gamma }h_{\gamma }^{\text{bk}}\delta {\tilde{c}}_{\gamma }\left(r\right)\;dr. \end{array}$$

Upon simple rearrangement, we can write

$$\begin{array}{ll} & \underset{\gamma }{\sum }\rho_{\gamma }\int_{V_{\text{cell}}}h_{\gamma }\left(r\right)\delta {\tilde{c}}_{\gamma }\left(r\right)\;dr=\frac{1}{2}\underset{\gamma }{\sum }\rho_{\gamma }\int_{V_{\text{cell}}}\delta \left[h_{\gamma }\left(r\right){\tilde{c}}_{\gamma }\left(r\right)-h_{\gamma }^{\text{bk}}{\tilde{c}}_{\gamma }\left(r\right)\right]\;dr, \end{array}$$ (13)

for which the left hand side now has the form of an exact differential.

Taking together Eqs. (10), (12), and (13), we can derive a final expression for the excess chemical potential as follows:

$$\begin{array}{l} \Delta \mu^{\text{HNC}}=\beta^{-1}\underset{\gamma }{\sum }\rho_{\gamma }\int_{V_{\text{cell}}}\frac{h_{\gamma }^{2}\left(r\right)}{2}-\left(1-\frac{h_{\gamma }^{\text{bk}}}{2}\right){\tilde{c}}_{\gamma }\left(r\right)-\frac{h_{\gamma }\left(r\right){\tilde{c}}_{\gamma }\left(r\right)}{2}\;dr, \end{array}$$ (14)

which is identical to the HNC expression for Eq. (1) except for the use of ${\tilde{c}}_{\gamma }\left(r\right)$ and an additional term, $-kT\sum_{\gamma }\rho_{\gamma }\int_{V_{\text{cell}}}-\frac{1}{2}h_{\gamma }^{\text{bk}}{\tilde{c}}_{\gamma }\;dr$, resulting from using the renormalized direct correlation function, ${\tilde{c}}_{\gamma }\left(r\right)$. This additional term will be present for all closures with a closed form expression of the excess chemical potential. A similar treatment for the PSE-*n* family of closures,^33^ which includes the Kovalenko–Hirata (KH) closure,^39^ is presented in the Appendix.

### D. Computing solvation forces on the periodic solute atoms

A closed form expression for the solvation force on atom *i* due to Eq. (14),

$$f_{i}\left(R_{i}\right)=\frac{\partial }{\partial R_{i}}\Delta \mu ,$$

may also be derived following the approach of Kovalenko and Hirata.^34^ For simplicity, we will again use the HNC expression for the excess chemical, Eq. (14), as the approach is easily extended to any closure with a closed form expression for the excess chemical potential. For example, the variation of Eq. (14) is given by

$$\delta \Delta \mu^{\text{HNC}}=kT\underset{\gamma }{\sum }\rho_{\gamma }\int_{V_{\text{cell}}}\left[h_{\gamma }\left(r\right)\delta h_{\gamma }\left(r\right)-\delta \left(\frac{h_{\gamma }\left(r\right){\tilde{c}}_{\gamma }\left(r\right)}{2}\right)\right.$$ (15)

$$\;\left.-\left(1-\frac{h_{\gamma }^{\text{bk}}}{2}\right)\delta {\tilde{c}}_{\gamma }\left(r\right)\;dr\right].$$ (16)

However, variations in the total and direct correlation functions are difficult to numerically compute, and we would like to confine the variation to the potential only. Solving for $g_{\gamma }\left(r\right)\beta \delta u_{\gamma }^{\text{PME}}\left(r\right)$ in Eq. (11) and simplifying, we have

$$g_{\gamma }\left(r\right)\beta \delta u_{\gamma }^{\text{PME}}\left(r\right)=h_{\gamma }\left(r\right)\delta h_{\gamma }\left(r\right)-h_{\gamma }\left(r\right)\delta {\tilde{c}}_{\gamma }\left(r\right)-\delta {\tilde{c}}_{\gamma }\left(r\right).$$

Using Eq. (13), we can write

$$\begin{array}{ll} & \underset{\gamma }{\sum }\rho_{\gamma }\int_{V_{\text{cell}}}g_{\gamma }\left(r\right)\beta \delta u_{\gamma }^{\text{PME}}\left(r\right)\;dr\\ & \;=\underset{\gamma }{\sum }\rho_{\gamma }\int_{V_{\text{cell}}}h_{\gamma }\left(r\right)\delta h_{\gamma }\left(r\right)-\delta \left(\frac{h_{\gamma }\left(r\right){\tilde{c}}_{\gamma }\left(r\right)}{2}\right)\\ & \;\;-\left(1-\frac{h_{\gamma }^{\text{bk}}}{2}\right)\delta {\tilde{c}}_{\gamma }\left(r\right)\;dr. \end{array}$$

As the right hand side matches the summation in Eq. (16), we have

$$\delta \Delta \mu^{\text{HNC}}=\underset{\gamma }{\sum }\rho_{\gamma }\int_{V_{\text{cell}}}g_{\gamma }\left(r\right)\delta u_{\gamma }^{\text{PME}}\left(r\right)\;dr.$$

Taking the variation with respect to the position of a solute atom, **R**_*i*_, we have

$$\begin{array}{ll} f_{i}\left(R_{i}\right) & =\frac{\partial }{\partial R_{i}}\Delta \mu^{\text{HNC}}\\ & =\underset{\gamma }{\sum }\rho_{\gamma }\int_{V_{\text{cell}}}g_{\gamma }\left(r\right)\frac{\partial }{\partial R_{i}}u_{\gamma }^{\text{PME}}\left(r\right)\;dr. \end{array}$$ (17)

This expression is the same as that for the standard 3D-RISM equation and independent of $h_{\gamma }^{\text{bk}}$.

## III. METHODS

### A. Solute preparation

With the exception of the heme group for myoglobin (PDB ID 1BZR) and guanosine-5'-triphosphate (GTP) in the hammerhead ribozyme (PDB ID 2QUS), all solvent and non-standard residues were removed from the deposited crystal structures. All protein and RNA structures were parameterized with the standard Amber charges and Lennard-Jones parameters,^52^ which have not changed since 1995. Naproxen was parameterized with the general Amber force field 2 (GAFF2).^53^ Parameters for hemoglobin^54^ and GTP^55^ were taken from the Amber contributed parameter database. The minimizations for 2OIU and 1Y0Q used the RNA ff99OL3 force field.^56,57^

### B. Solvent preparation

The properties of the bulk solvent, including ${\hat{\chi }}_{\alpha \gamma }\left(k\right)$, required for Eq. (8) were precomputed with *rism1d* from AmberTools 21.^12,58^ In all cases, the dielectrically consistent RISM (DRISM)^24^ was solved at a temperature of 298 K with a dielectric constant of 78.497 and the KH closure^39^ on a grid with a spacing of 0.025 Å and 32  768 points to a residual tolerance of 10^−12^. The coincident extended simple point charge model (cSPC/E) water model was used with Joung–Cheatham parameters for monovalent ions^59^ and Li-Merz 12-6 parameters for divalent ions.^60^ The details of the solvent composition for each solute can be found in Table I.

**TABLE I.** 3D-RISM parameters for crystal structure optimization and energy minimization. The grid spacing is in Å.

PDB/CSD ID	Spacing	Grid size	Solvent
ANOMEW^40^	0.33	84 × 72 × 96	0.005M MgCl_2(*aq*)_
1AHO^41^	0.4	120 × 108 × 80	Water
2IGD^42^	0.35	108 × 120 × 126	Water
1BZR^43^	0.35	108 × 190 × 192	Water
4LZT^44^	0.35	80 × 96 × 108	Water
2LZT^45^	0.35	80 × 96 × 108	Water
4YUL^46^	0.35	126 × 160 × 256	Water
2A43^47^	0.35	160 × 160 × 160	0.02M MgCl_2_, 0.14M KCl_(*aq*)_
480D^48^	0.35	90 × 90 × 224	1M NaCl_(*aq*)_
2QUS^49^	0.35	80 × 160 × 210	1M NaCl_(*aq*)_
1Y0Q^50^	1.0	96 × 144 × 224	0.02M MgCl_2_, 0.14M KCl_(*aq*)_
2OIU^51^	1.0	48 × 112 × 80	0.1M MgCl_2_, 1.29M NaCl_(*aq*)_

### C. 3D-RISM calculations

Equation (8) was solved using *sander* from AmberTools 21, modified as described in Sec. II. Except where described in Sec. IV, grid sizes and spacings are detailed in Table I. Calculations requiring solvation forces (Secs. IV A and IV D) were solved to a residual tolerance of 10^−10^, while all other calculations were solved to a residual tolerance of 10^−7^. For biomolecular crystals, grid dimensions were selected to match the unit cells of the deposited structures, with exceptions noted for the calculations discussed in Secs. IV A and IV B. For the small molecule crystal naproxen calculation, the original unit cell was expanded 3, 7, and 3 times, respectively, along the three crystal lattice vectors.

## IV. RESULTS

We have applied this periodic 3D-RISM model to a variety of protein and nucleic acid crystals. We begin with discussions of the accuracy of forces on solute atoms arising from the gradients of the excess chemical potential (Sec. IV A) and then look at the way a periodic system extrapolates to a non-periodic limit as the size of the periodic box surrounding a single solvent molecule increases (Sec. IV B). These help to provide confidence in the correctness of our implementation. We then look at examples of the solvent distributions in biomolecules, comparing x-ray scattering factors (Sec. IV C), and give examples of predictions for electrostatic screening effects in RNA crystals (Sec. IV D). These show promising results, but it is clear that many more studies will be needed to map out the expected level of accuracy of this approach.

### A. Accuracy of atomic forces

The use of the 3D-RISM as an implicit solvent requires accurate and rapid calculation of atomic forces. Both speed and accuracy may depend upon the system. Table II gives some results for a small RNA unit cell, with 108 nucleotides in four chains. We compare gradients computed via Eq. (17) to those computed with finite differences using Eq. (14). There is smooth convergence with respect to grid spacing for both Δ*μ* and the accuracy of the gradients, but very large grids can be expensive. For the practical examples discussed in Sec. IV D, we find that a grid spacing of 0.5 Å gives results that hardly differ from tighter grids. This is supported by the numbers of excess water molecules and ions presented in Table II, which show that the key properties of the solvent distribution are converged even at the larger grid spacings. The actual value of Δ*μ* is not available from the experiment, so grid artifacts in estimating its value are of little consequence provided that the gradients and solvent distributions are accurate. This appears to be the case for even the largest grid spacings shown in this table.

**TABLE II.** Comparison of gradients computed via Eq. (A1) to those computed with finite differences using a displacement of 10^−4^ Å. The grid spacing is in Å. MAE is the mean absolute error, and max is the maximum absolute error (both in kcal/mol-Å) for the *x*, *y*, and *z* components of the gradient for 20 randomly selected atoms. Δ*μ* is the excess chemical potential in kcal/mol. The final four columns give the excess number of water molecules and ions. The system is one unit cell of the sarcin–ricin system PDB ID 480d, with 108 nucleotides and a solute charge of −104*e*. The solvents are 0.02M MgCl_2_ and 0.14M KCl in water.

Grid spacing	MAE	Max	Δ*μ*	H_2_O	Mg^2+^	K^+^	Cl^−^
0.75	0.0053	0.0332	82.7	−1049.6	19.89	56.79	−7.43
0.50	0.0026	0.0128	54.2	−1047.6	19.89	56.79	−7.43
0.25	0.0006	0.0040	43.9	−1047.3	19.89	56.79	−7.43
0.15	0.0004	0.0018	43.6	−1047.2	19.89	56.79	−7.43

It is worth noting that the “additional” background contribution of $-kT\sum_{\gamma }\rho_{\gamma }\int_{V_{\text{cell}}}-\frac{1}{2}h_{\gamma }^{\text{bk}}{\tilde{c}}_{\gamma }\;dr$ in Eqs. (14) and (A1) is key for periodic calculations. If this contribution is omitted, the value of Δ*μ* changes to −468 kcal/mol (for a grid spacing of 0.5 Å), and the mean and maximum absolute derivative errors are 0.47 and 1.86 kcal/mol-Å, respectively, more than two orders of magnitude larger than the values shown in Table II.

By comparison, for a single solute in a large box, this “additional” term is quite small. As an example, consider one chain of sarcin–ricin from Table II. Even with a fairly large solute charge of −26*e*, embedding this in a 120 Å box yields Δ*μ* values of −5941.41 kcal/mol without the “correction” and −5941.62 with it, with a difference of 0.21 kcal/mol.

### B. Extrapolation to the infinite dilution regime

The examples discussed above dealt with molecular crystals, where solute molecules are in contact with their images in neighboring unit cells, and the solvent volume is fairly small. Another application might be to a single (dilute) solute surrounded by a buffer of solvent. As the size of the unit cell increases, such a calculation should approach the infinite dilution, the non-periodic limit that has traditionally been assumed in 3D-RISM applications. As noted above, these traditional calculations actually employ a regular periodic grid in the vicinity of the solute (to enable convolutions to be carried out via the fast Fourier transform) and add in estimates of the “asymptotic” contributions from the solvent outside the grid. Here, we study the box-size dependence of periodic 3D-RISM calculations that have a single solute molecule at the origin.

**FIG. 2.**
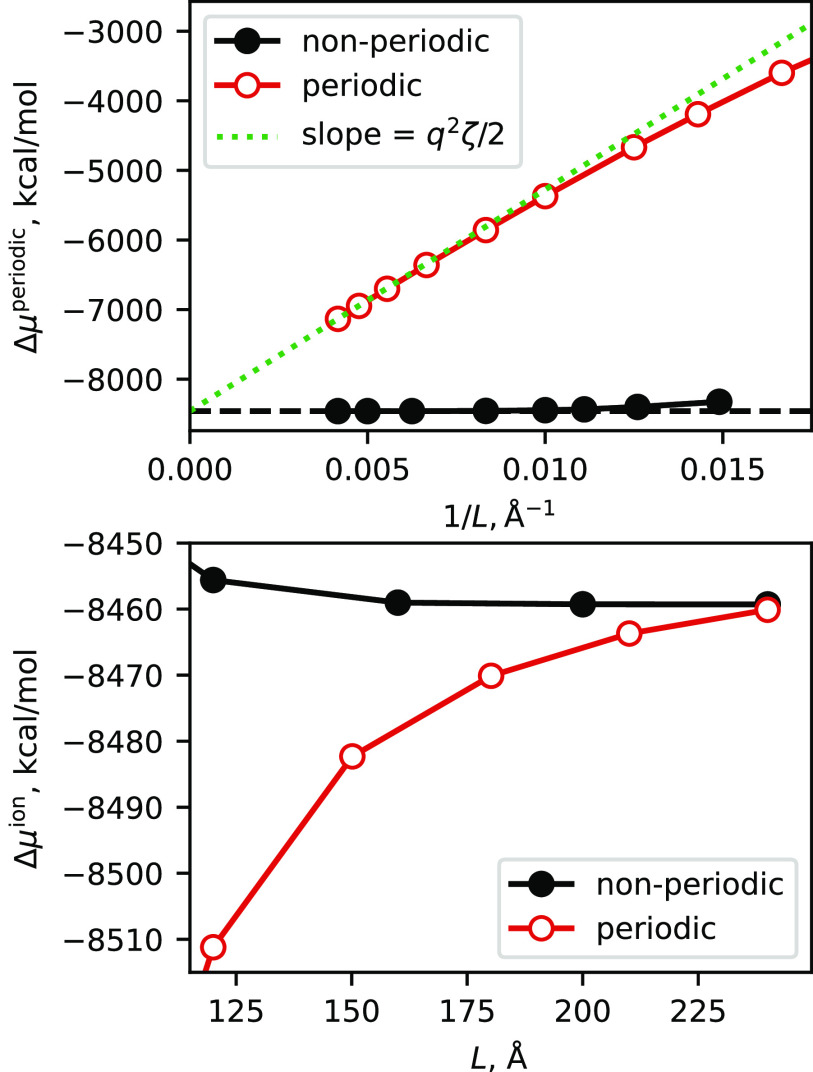
Variation of solute excess chemical potential with respect to cell size. A single sarcin–ricin RNA chain, taken from PDB ID 480d, is placed in cubic boxes of varying sizes. The solvent is 0.1M NaCl in water, with a grid spacing of 0.5 Å. Top: original results, plotting Δ*μ*^periodic^; the green line has a slope of *q*^2^*ζ*/2. Bottom: the periodic result is corrected to Δ*μ*^ion^ via Eq. (18) and shown for large box sizes.

The thermodynamic quantity of most direct interest is the excess chemical potential, Δ*μ*, since this (when added to the potential energy of the solute alone) creates the potential of mean force that is used when applying the 3D-RISM as an implicit solvent model. As discussed above, for a solute with a net charge, the periodic model we use has a uniform background charge to neutralize the system. A periodic system with charged molecules and such a uniform background charge is often called a “Wigner lattice,” and the effects of periodicity can be computed and removed in order to facilitate comparison to analogous non-periodic calculations. For a cubic cell, the result for a single ion, Δ*μ*^ion^, is related to the periodic result as follows:^61^

$$\Delta \mu^{\text{ion}}=\Delta \mu^{\text{periodic}}-q^{2}\zeta /2L,$$ (18)

where *q* is the net charge on the solute, *L* is the box length, and *ζ* = 2.837. Figure 2 shows results for a 27-nucleotide RNA stem-loop with a net solute charge *q* of −26*e*. The comparison is to parallel calculations with the existing non-periodic 3D-RISM codes in Amber. The top plot illustrates the near-linear dependence on 1/*L* expected from Eq. (18); the lower plot directly compares Δ*μ*^ion^ for periodic and non-periodic codes. In the limit of large box sizes, the two results converge to the same value (to within 1 kcal/mol at *L* = 240 Å), but the non-periodic code is much less sensitive to box size. This is expected since the non-periodic result includes an “asymptotic” contribution that estimates contributions beyond the box used for the convolution; this is quite an accurate estimate that provides reasonably converged results even for modest box sizes. For this reason, the use of the periodic code for non-periodic problems is not an attractive option, at least at present. Nevertheless, the existing non-periodic codes have been well tested for many types of problems, and the convergence illustrated in Fig. 2 provides evidence for the correctness of the new periodic implementation.

Another feature of interest, beyond thermodynamics, lies in the solvent distribution itself. Quantities such as the excess number of ions (or water molecules) around a charged solute can be measured experimentally^1,2,4,62^ and compared with computations. These distributions converge much more quickly with box size or grid spacing than does Δ*μ* itself. Table II gives such values for the sarcin–ricin RNA in a mixed salt with Mg^2+^, K^+^, and Cl^−^ ions. Going from a grid spacing of 0.75 Å to 0.25 Å changes Δ*μ* by 39 kcal/mol, whereas the excess number of ions changes hardly at all, even the excess number of water molecules changes by only 0.2%.

### C. Solvent distributions in small molecule crystals

**FIG. 3.**
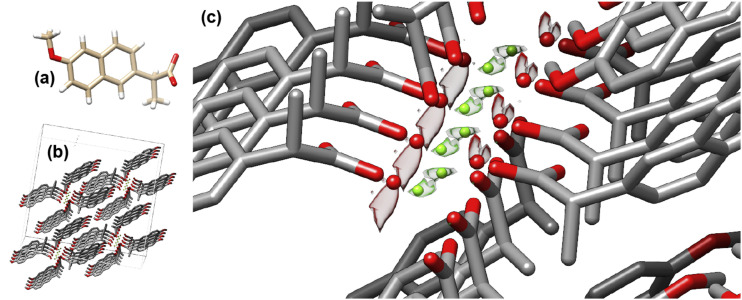
(a) Chemical structure and (b) crystal structure of naproxen H_2_O·Mg^2+^. (c) Solvent density contours for oxygen from water [red, $g_{\text{O}}\left(r\right)=30.0$] and Mg^2+^ [green, $g_{{\text{Mg}}^{2+}}\left(r\right)=4000.0$], with colored spheres showing the locations of localized water molecules and ions as assigned in the refinement process. The large values of the pair distribution function at the contour surface indicate that the water oxygen atoms and magnesium ions are highly localized. In the case of magnesium, the value is particularly large due to its low concentration in the bulk liquid (Table I).

One of the key advantages of an atom-based solvent model, such as the 3D-RISM, compared to continuum implicit solvent models, is that a thermally averaged solvent distribution profile (on a 3D-grid) is available for each solvent component. A simple small-molecule example is the non-steroidal anti-inflammatory drug naproxen whose crystal structure (CCDC entry ANOMEW^40^) as a hydrate with water and Mg^2+^ is shown in Fig. 3. The solvent density contours from 3D-RISM closely match the electron density distributions from x-ray crystallography. This may not be surprising in this case since the solvent channel is narrow but offers prospects for the analyses of many polymorphs of naproxen that have different amounts of water molecules and cations, sometimes with clear evidence of the disordered solvent. Similar predictions are available for biomolecules, such as for the RNA crystals discussed below, but there it is more difficult to evaluate the accuracy of the 3D-RISM results since only a small percentage of the ions and water molecules that must be present in the crystal can be located in electron density maps.

One way to evaluate the quality of the predicted solvent distributions is to use them (in combination with atomic models for the solute molecules) to compute x-ray scattering intensities that can be compared to those observed from x-ray crystallography. Since atomic models for macromolecules almost never reproduce experimental x-ray scattering amplitudes to within experimental data (a feature that is sometimes called the “R-factor gap”^11^), we compare results using the 3D-RISM with the standard “flat” solvent models employed in conventional crystallographic refinement.

**FIG. 4.**
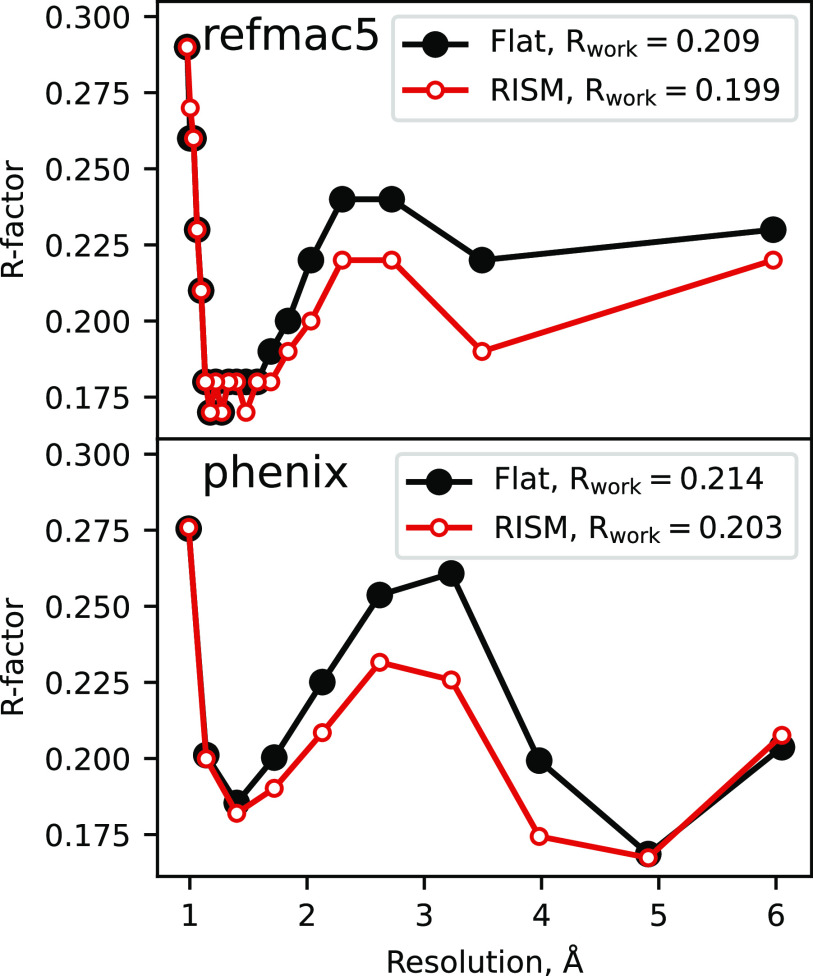
Refinement of 1AHO^41^ in *refmac*5 (top) and *phenix* (bottom) using a flat solvent density model and 3D-RISM.

The results are shown in Fig. 4 and Tables III and IV. Refinement calculations were performed using two popular macromolecular refinement codes, *refmac*5^63^ and *phenix*.^64^ These two codes give broadly similar results but differ in details of how the flat solvent model is implemented and how reflections are binned by resolution and subsequently scaled. The 3D-RISM solvent density maps were computed using the deposited solute atomic models (keeping only the most highly occupied alternate conformations) with solvent molecules removed. During refinement, the solvent density is held constant (except for overall scaling and overall B-factors, which are refined), and the atomic positions and B-factors of the solute are modified to achieve the best agreement with the observed diffraction intensities. We used 40 refinement cycles for *refmac*5 starting from the deposited solute atomic model. Parallel refinements were carried out using the default, “flat,” solvent density model. The *phenix.refine* package does not have a fully comparable capability, but we can compare the 3D-RISM and flat bulk-solvent models for the deposited solute atomic model.

**TABLE III.** Bulk solvent models with a single protein configuration; each block shows R/Rfree after 40 cycles of *refmac*5 refinement.

Protein	Scorpion-toxin	GB3	Myoglobin	Lysozyme	Lysozyme	Cyclophilin
PDB ID/resol.	1AHO/0.96	2IGD/1.10	1BZR/1.15	4LZT/0.95	2LZT/1.97	4YUL/1.42
Flat (refmac)	0.209/0.214	0.220/0.233	0.200/0.208	0.196/0.205	0.167/0.216	0.201/0.224
3D-RISM	0.199/0.211	0.213/0.224	0.194/0.206	0.190/0.197	0.154/0.201	0.185/0.202

**TABLE IV.** Bulk solvent models with a single RNA configuration; each block shows R/Rfree after 40 cycles of *refmac*5 refinement.

RNA	Pseudoknot	Sarcin–ricin loop	Hammerhead
PDB ID/resol.	2A43/1.34	480D/1.50	2QUS/2.40
Flat (refmac)	0.223/0.261	0.192/0.216	0.206/0.255
3D-RISM	0.208/0.229	0.175/0.208	0.186/0.234

Figure 4 shows results for a 64-residue scorpion toxin protein, PDB code 1AHO. There is an overall drop of about 1% between the flat and 3D-RISM solvent models, with about a 2% improvement in resolutions between 2 and 4 Å, whereas there is little difference at lower and higher resolutions. This is not an insignificant improvement (given that there are no new adjustable parameters) and provides a benchmark example for other solvent models, such as those based on other closures or on MD simulations: better solvent models should yield lower R-factors. For now, this calculation only provides better “statistics;” this solvent model would need to be integrated into a refinement algorithm to see what effect it would have on the final atomic model. (Such studies will be reported elsewhere.) It is likely that improved models may involve some combination of explicit water molecules (placed into locations identified in the electron density map) and a 3D-RISM model for the remaining (“disordered” or “bulk”) solvent. These more complex models have more adjustable parameters, which will have to be balanced against improvements in the resulting R-factors.

Tables III and IV show overall drops in R and Rfree for a selection of small proteins and RNA crystals. In each case, R and Rfree are improved: on average, the 3D-RISM values for Rfree are 1.3% better than when using the default flat solvent model in *refmac*5.

### D. Using 3D-RISM as an implicit solvent model for biomolecular crystals

In addition to providing a map of the distribution of solvent molecules in the crystal lattice, the integral equation approach provides a solvation free energy and its gradients with respect to solute atomic positions. This provides an implicit solvent model that can be used for minimizations or molecular dynamics. This has been found to work well in non-periodic situations (e.g., for DNA^65,66^), giving results that are often superior to numerical Poisson–Boltzmann or generalized Born models.^67^ Since there are a very few implicit solvent models that work for crowded periodic systems, such as molecular crystals, this is an intriguing approach despite its relatively high computational cost.

**FIG. 5.**
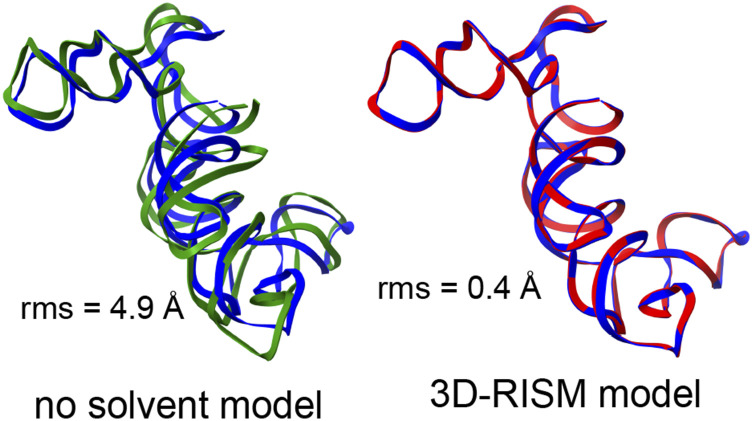
Blue: experimental structure from x-ray crystallography (PDB ID 2OIU), red: structure from a 3D-RISM crystal minimization, and green: structure from a crystal minimization with no solvent correction. rms gives the root-mean-square deviation (in Å) of all non-hydrogen atoms from the crystal structure. Only a single chain is shown, but the calculation included the entire unit cell.

**FIG. 6.**
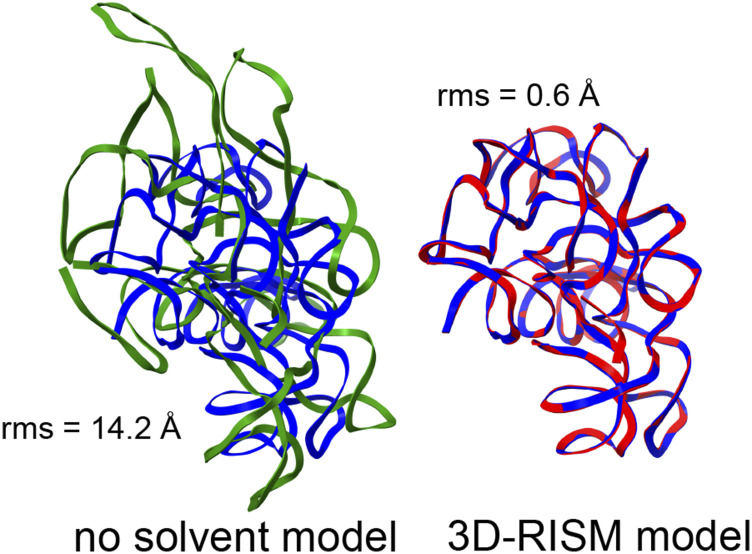
Same as Fig. 5 but for the PDB code 1Y0Q.

The need to include the energetic aspects of solvation is especially important for nucleic acid crystals where there are many charged phosphate groups in close proximity, and generally, only a small number of counter ions are visible in the electron density maps. We consider two examples here: the L1 ribozyme ligase circular adduct (PDB code 2OIU^51^) and a group I intron product complex (PDB code 1Y0Q^50^). Figures 5 and 6 show the results of minimization calculations in the crystal lattice, with and without the 3D-RISM implicit solvent model. For the smaller 2OIU system (9188 solute atoms), we carried out 1100 steps of conjugate gradient minimization (using the LBFGS algorithm), followed by 30 steps of truncated-Newton conjugate gradient optimization. The root-mean-square (rms) of the elements of the final gradient was 0.02 kcal/mol-Å, and the energy drop on the final step of truncated-Newton optimization was 0.3 kcal/mol. For 3D-RISM with a grid spacing of 1.0 Å, each energy evaluation took 13 s using 16 MPI threads on a single Xeon Gold 6230 CPU running at 2.10 GHz. The larger 1Y0Q system (60 288 solute atoms) was minimized for 400 steps of conjugate gradient minimization, with a final rms gradient of 0.02 kcal/mol. Here, each energy evaluation required 9 min on 16 threads on a single CPU.

Figures 5 and 6 show the superpositions of a single RNA chain even though the simulations themselves included a full unit cell that is periodically replicated. In both examples, it is clear that the lack of solvent screening of the phosphate–phosphate interactions in the “no solvent model” minimizations results in an expansion of the system, even within the constraints of the crystal lattice, whereas the 3D-RISM calculations show excellent fidelity to the experimental structural models. (It is not enough to just reduce the net charge on phosphate groups: for 1Y0Q, a “vacuum” minimization where the net charge on each phosphate is reduced from −1.0 to −0.2, in rough accord with counterion condensation models, still results in an rms shift of 4.7 Å.) In a refinement calculation without the implicit solvent model, the force-field energies would be fighting against the x-ray restraints, whereas the results of Figs. 5 and 6 suggest that this would be much less true if 3D-RISM were employed.

The fairly slow timings for 3D-RISM will limit some potential applications but need not impede useful results. For example, a typical ten-cycle refinement run in the *phenix* package of programs^64^ typically makes fewer than 300 energy evaluations during the coordinate refinement steps so that even a system as large as 1y0q would need less than 2 days, which is not inappropriate for a final refinement step. (We have begun coding a graphics processing unit (GPU)-enabled version of these models and hope that this will provide a significant speed improvement over the CPU results reported here.)

As an example, we show in Table V results for several crystallographic refinement calculations for the group I intron, PDB code 1Y0Q. The diffraction data here are only at a resolution of 3.6 Å, so many structural details are not well-determined by the x-ray data alone. The first column shows the deposited results and gives statistics from the *MolProbity* program.^68^ The next two columns show parallel refinements (starting from the deposited structure) using phenix: the “phenix_cdl” column uses the default geometric restraints from its conformational dependent library, which are largely similar to conventional Engh–Huber restraints. The “phenix_amber” column replaces the cdl restraints with forces from the Amber force field, as described elsewhere.^70^ This force field model has no implicit solvent contribution and hence no charge-screening effects. The final column adds in the 3D-RISM model as in Fig. 6; we used in-house codes to carry out the coordinate refinements and *phenix.refine* for isotropic B-factor refinements, alternating cycles of 150 refinement steps of coordinate refinement with five macro-cycles of B-factor optimization.

**TABLE V.** Results for several test refinements of 1Y0Q. The first seven rows come from the MolProbity program;^68^ the root-mean-square (rms) change from the deposited structure is computed for all non-hydrogen atoms. Some results are reproduced with permission from J. G. Gray and D. A. Case, Crystals **11**, 771 (2021). Copyright 2021 Author(s), licensed under a Creative Commons Attribution (CC BY) license.^69^

	1Y0Q	phenix_cdl	phenix_amber	3D-RISM
ClashScore	53.7	35.4	3.7	0.9
rms (bonds)	0.008	0.011	0.017	0.015
rms (angles)	1.35	2.10	3.00	2.00
MolProbity score	3.35	3.18	2.31	1.91
Pucker outliers (%)	8.6	8.6	10.7	8.2
Angle outliers (%)	0.7	0.7	9.4	2.0
Average suiteness	0.492	0.414	0.307	0.574
R-work	0.277	0.221	0.264	0.251
R-free	0.310	0.278	0.307	0.293
rms from deposition	0.00	0.36	0.71	0.37

The overall results are in general agreement with earlier studies on proteins.^70^ The use of a force field greatly reduces the number of bad contacts, as evidenced by the ClashScore and improves the overall MolProbity score. However, the RNA-specific scores for sugar pucker, sugar angles, and “suiteness” (a measure of how well sugar-phosphate torsion angles agree with databases of well-refined structures) get worse in the phenix_amber results. This is presumably because the force field itself prefers an expanded structure (Fig. 6) and its gradients compete with those from the observed structure factors. The addition of the 3D-RISM model improves all the structural features and reduces the shift away from the deposited structure. The comparable results for six additional RNA crystals are presented elsewhere.^69^

It is clear that many more studies will be needed to establish the generality of these results: in proteins, where charge screening effects are less important, more than 13 000 such parallel refinements were carried out to help establish the expected behavior.^70^ Systems with higher-resolution diffraction data should depend less on the nature of the geometric restraints than do lower-resolution structures. However, these initial results illustrate what is now possible in this regard.

## V. CONCLUSIONS

Water molecules and ions around biomolecules often play a crucial role in the function. The analysis of the solvent distributions in biomolecular crystals can provide an important check on the accuracy of computational models. Here, we present an implementation of the 3D-RISM solvent model that can be applied to any periodic system, including “crowded” systems, such as crystals, where the majority of space is taken up by the solute.

In many ways, the periodic version is not a major departure from existing non-periodic 3D-RISM codes since a fast Fourier transform (with a periodic cell) has always been used to compute the convolutions needed for the Ornstein–Zernike equation. The machinery to compute the periodic potential energy was adapted from existing particle-mesh-Ewald (PME) procedures in the molecular dynamics code. However, a key advance was required for charged solutes: a modification of the total correlation function *h* is needed [Eq. (9)] to account for the implicit neutralizing potential arising from the PME procedure, and this, in turn, implies an extra contribution to the excess chemical potential [Eq. (14)] that had not been recognized before. This contribution is negligible for non-periodic systems but can become important for crowded crystalline environments. With this correction, analytical expressions for forces on the solute atoms closely match gradients computed by the finite difference, and the periodic expressions smoothly merge to existing non-periodic results for a single solute as the size of the periodic cell increases. Our approach for charged solutes does involve a uniform background charge distribution (so that *u*^PME^ can be used in place of *u*). This method of unit-cell neutralization is neither physical nor unique but does lead to an internally consistent approach with accurate gradients (Table II) and preliminary results that are promising even for highly charged systems (Figs. 5 and 6 and Table V).

It is clear that much effort will be required to understand the expected accuracy of this approach and that improvements in potentials and in closure relations should be examined. The predicted solvent distributions can be compared to the experiment in a variety of ways: by looking at the locations of ordered water molecules and ions that can be identified in density maps derived from x-ray crystallography, by comparing computed and observed Bragg intensities, and (potentially) by comparing predicted and measured crystal densities (which reflect the total number of water molecules and ions per unit cell). The use of 3D-RISM as a periodic implicit solvent model can be tested by molecular dynamics or minimization calculations in cases where experimental structures are available. We have provided a few examples of such comparisons here, but many more are needed. Improvements in efficiency will help to make this a practical method; porting the codes to a GPU environment is under way.

The periodic 3D-RISM implementation used here will be included in AmberTools, an open source collection of molecular simulation software, and may be downloaded at https://ambermd.org. The implementation was based upon an existing non-periodic RISM code that was primarily developed by Luchko *et al.*^12^ Extensions to periodic systems were implemented by Jesse Johnson and George Giambasu, and a more complete description of the codes is given elsewhere.^38^

## Data Availability

The data that support the findings of this study are available from the corresponding author upon reasonable request and can be reproduced with the AmberTools 21 software suite.^58^

## APPENDIX: EXCESS CHEMICAL POTENTIAL FOR THE PSE-n CLOSURE FAMILY

For the partial series expansion of order-*n* (PSE-*n*) family of closures,^33^ which includes the Kovalenko–Hirata (KH) closure,^39^ we have

$$g_{\gamma }\left(r\right)=\left\{\begin{array}{cc} \overset{n}{\underset{0}{\sum }}\frac{{\left(t_{\gamma }^{*}\left(r\right)\right)}^{i}}{i!},\; & t^{*}\left(r\right)>0,\\ \exp\left(t^{*}\right),\; & t^{*}\left(r\right)\leq 0, \end{array}\right.$$

which has the bridge function

$$B_{\gamma }\left(r\right)=\left\{\begin{array}{cc} -t_{\gamma }^{*}\left(r\right)+\ln\left(\overset{n}{\underset{0}{\sum }}\frac{{\left(t_{\gamma }^{*}\left(r\right)\right)}^{i}}{i!}\right),\; & t^{*}\left(r\right)>0,\\ 0,\; & t^{*}\left(r\right)\leq 0, \end{array}\right.$$

where $t_{\gamma }^{*}\left(r\right)=-\beta u_{\gamma }\left(r\right)+h_{\gamma }\left(r\right)-c_{\gamma }\left(r\right)$. Because we have a non-zero bridge function, we must consider the variation in the general form of the closure, Eq. (2),

$$\begin{array}{ll} \delta h_{\gamma }\left(r\right) & =-g_{\gamma }\left(r\right)\beta \delta u_{\gamma }^{\text{PME}}\left(r\right)+g_{\gamma }\left(r\right)\delta h_{\gamma }\left(r\right)\\ & \;-g_{\gamma }\left(r\right)\delta {\tilde{c}}_{\gamma }\left(r\right)+g_{\gamma }\left(r\right)\delta B_{\gamma }\left(r\right). \end{array}$$

All but the last was considered in Sec. II C. For the last term, we have an exact differential

$$\begin{array}{ll} g_{\gamma }\left(r\right)\delta B_{\gamma }\left(r\right) & =\left\{\begin{array}{cc} \overset{n}{\underset{0}{\sum }}\frac{{\left(t_{\gamma }^{*}\left(r\right)\right)}^{i}}{i!}\left[-\delta t_{\gamma }^{*}\left(r\right)\right.\;\\ \left.+\frac{1}{\overset{n}{\underset{0}{\sum }}\frac{{\left(t_{\gamma }^{*}\left(r\right)\right)}^{i}}{i!}}\overset{n-1}{\underset{0}{\sum }}\frac{{\left(t^{*}\left(r\right)\right)}^{i}}{\left(i\right)!}\delta t_{\gamma }^{*}\left(r\right)\right],\; & t^{*}\left(r\right)>0,\\ 0,\; & t^{*}\left(r\right)\leq 0 \end{array}\right.\\ & =-\delta \frac{{\left(t_{\gamma }^{*}\left(r\right)\right)}^{n+1}}{\left(n+1\right)!}\Theta \left(t_{\gamma }^{*}\left(r\right)\right), \end{array}$$

where $\Theta \left(\right)$ is the Heaviside function, and we have used $\delta {\left(t_{\gamma }^{*}\left(r\right)\right)}^{n}=nt_{\gamma }^{*}{\left(r\right)}^{n-1}\delta t_{\gamma }^{*}\left(r\right)$. Using this result with Eqs. (10), (12), and (13), we have

$$\begin{array}{ll} \Delta \mu^{\text{PSE}-n} & =kT\underset{\gamma }{\sum }\rho_{\gamma }\int_{V_{\text{cell}}}\frac{{\left(h_{\gamma }\left(r\right)\right)}^{2}}{2}-\left(1-\frac{h_{\gamma }^{\text{bk}}}{2}\right){\tilde{c}}_{\gamma }\\ & \;-\frac{h_{\gamma }\left(r\right){\tilde{c}}_{\gamma }\left(r\right)}{2}-\frac{{\left(t_{\gamma }^{*}\left(r\right)\right)}^{n+1}}{\left(n+1\right)!}\Theta \left(t_{\gamma }^{*}\left(r\right)\right)\;dr. \end{array}$$ (A1)

As with the HNC closure, this expression is the same as the usual expression^33^ except for an additional term of $-kT\sum_{\gamma }\rho_{\gamma }\int_{V_{\text{cell}}}-\frac{1}{2}h_{\gamma }^{\text{bk}}{\tilde{c}}_{\gamma }\;dr$.
